# The mortality rates and the space-time patterns of John Snow’s cholera epidemic map

**DOI:** 10.1186/s12942-015-0011-y

**Published:** 2015-06-17

**Authors:** Narushige Shiode, Shino Shiode, Elodie Rod-Thatcher, Sanjay Rana, Peter Vinten-Johansen

**Affiliations:** Centre for Interdisciplinary Methodologies, University of Warwick, Coventry, CV4 7AL UK; Department of Geography, Environment and Development Studies, Birkbeck College, University of London, Malet Street, London, WC1E 7HX UK; Department of History, Michigan State University, East Lansing, MI 48824-1036 USA

**Keywords:** Cholera, Historical data, John Snow, Mortality rate, Population at risk, Spatial-temporal analysis

## Abstract

**Background:**

Snow’s work on the Broad Street map is widely known as a pioneering example of spatial epidemiology. It lacks, however, two significant attributes required in contemporary analyses of disease incidence: population at risk and the progression of the epidemic over time. Despite this has been repeatedly suggested in the literature, no systematic investigation of these two aspects was previously carried out. Using a series of historical documents, this study constructs own data to revisit Snow’s study to examine the mortality rate at each street location and the space-time pattern of the cholera outbreak.

**Methods:**

This study brings together records from a series of historical documents, and prepares own data on the estimated number of residents at each house location as well as the space-time data of the victims, and these are processed in GIS to facilitate the spatial-temporal analysis. Mortality rates and the space-time pattern in the victims’ records are explored using Kernel Density Estimation and network-based Scan Statistic, a recently developed method that detects significant concentrations of records such as the date and place of victims with respect to their distance from others along the street network. The results are visualised in a map form using a GIS platform.

**Results:**

Data on mortality rates and space-time distribution of the victims were collected from various sources and were successfully merged and digitised, thus allowing the production of new map outputs and new interpretation of the 1854 cholera outbreak in London, covering more cases than Snow’s original report and also adding new insights into their space-time distribution. They confirmed that areas in the immediate vicinity of the Broad Street pump indeed suffered from excessively high mortality rates, which has been suspected for the past 160 years but remained unconfirmed. No distinctive pattern was found in the space-time distribution of victims’ locations.

**Conclusions:**

The high mortality rates identified around the Broad Street pump are consistent with Snow’s theory about cholera being transmitted through contaminated water. The absence of a clear space-time pattern also indicates the water-bourne, rather than the then popular belief of air bourne, nature of cholera.

The GIS data constructed in this study has an academic value and would cater for further research on Snow’s map.

## Background

In the fields of epidemiology, public health and medical geography, John Snow is often referred to as ‘hero of cholera’ [1] for his seminal work in creating a map of cholera deaths in Soho, London [2] (although some report that the exact nature of his contribution and achievement is debatable. Please refer to [1] and [3] for more details). The locations of the cholera victims drawn on his map confirmed his theory about the disease being transmitted through contaminated water, contrary to the popular belief at the time that it was airborne [4]. The map commonly known as Snow’s cholera map consists of a base map originally drawn by a renowned mapmaker Charles F. Cheffins on which Snow added a total of 578 victims’ locations and an equidistance line around the areas served by the Broad Street pump (hereafter called the ‘BSP’) as their closest water source [4]. It clearly illustrates the concentration of victims around the BSP location, which served as an effective means for Snow to communicate his results to the Cholera Inquiry Committee and it is for this reason that his work has been seen as a pioneering example in the field of spatial epidemiology.

However, Koch [5] points out that Snow’s contribution remains largely visual and iconic, thus restricting the utility of his approach for detecting and monitoring disease outbreaks. For one thing, disease outbreaks are usually studied in relation to the population at risk, but Snow’s account of the cholera deaths was based on raw counts. In fact, despite the numerous studies carried out on his data since then, no systematic investigation has been yet carried out on the distribution of residential population in Soho at the time of the outbreak [6]. Investigating the distribution of population in the area will help confirm whether the concentration of victims around the BSP was indeed caused by high mortality rates, rather than a product of having a high number of residents in the area.

The other factor that demands attention is disease surveillance, or the understanding of how the epidemic spreads in an area over time. If cholera was indeed airbourne, then we would expect to see some form of spatial diffusion from a source, whereas a water-bourne disease is less likely to produce a clear pattern of diffusion over space and time. Studying such space-time patterns require information about *where* the deaths were recorded as well as *when* they were observed [7].

Despite the popularity of Snow’s map, these two factors, namely the mortality rate and the space-time signature of the epidemic in the affected area, are yet to be investigated. This study pursues these two aspects of the 1854 cholera outbreak. It constructs population data by combining street-address data from several different sources including historical Census as well as records on individual households found in medical reports on house-to-house visitations. This is compared against the distribution of victims so as to examine if areas around the BSP truly suffered from higher mortality rates. The degree of concentration of victims is measured through the application of a network-based cluster detection technique, which allows us to identify spatial clusters using the street-network distance. In order to facilitate spatial-temporal analysis of the epidemic, victims’ date of deaths are also added to the original data, along with additional cholera deaths identified in historical documents other than Snow’s report. This new dataset is analysed using two different types of cluster-detection methods: the traditional Kernel Density Estimation (hereafter called ‘KDE’) and the recently developed Network-based Scan Statistics (hereafter called ‘NetScan’) [8]. These methods are explained in a later section.

## Literature review

Snow’s data and map became widely known after Sedgwick first introduced it in the United States as a pioneering example of epidemiology in varying editions of his 1901 text “Sanitary Science” [1]. They were subsequently used widely by many, including W.H. Frost in 1936, as a case study that demonstrates the spatial distribution of a disease [1]. This tendency has been particularly prominent in recent years [4–6]. These studies can be broadly categorised into two strands of research: one that pursues the cartographic aspect of his map, and the other that is driven by a spatial analytical interest in his data.

### Cartographic pursuit of Snow’s work

Snow’s map has attracted strong interest in the field of cartography, primarily for its visual impact on demonstrating the concentration of cholera victims in the vicinity of the BSP [5]. The map has been cited by many in their work that focused on displaying the data effectively, and has been often adopted as an early example of effective visual communication. These include the work by Gilbert [9] and Monmonier [10] who reproduced Snow’s map using different cartographic elements, as well as those who offered a more historical account of how the map was produced in relation to other maps created at the time and why this particular map has acquired fame [4, 11].

Others have discussed the limitations of Snow’s map. For instance, Koch [3] pointed out that Snow’s map did not incorporate the uneven distribution of population density in the study area, thus failing to account for the population at risk. Around the time of the outbreak, St James was the most-densely populated of the 135 registration sub-districts in Greater London with 30 houses per acre, and an average of 10 persons per house; while other streets in the area had a lower population density [3].

### Spatial analytical pursuit of Snow’s work

In addition to the cartographic exploration of his map, Snow’s data have also attracted strong academic interests, as they provide an excellent material for spatial analyses. Most of these studies have been carried out with the aim to reproduce, test and develop Snow’s work. In particular, there are two groups of studies: one that takes an inductive approach to confirm BSP’s influence on the victim locations, and the other that focuses on a deductive approach of deriving the concentration of victim locations to infer the association with BSP’s location.

The first strand of analytical studies focuses on BSP’s area of influence, in relation to that of other pumps, to examine if the victims’ locations would indeed fall within that area. These studies were carried out mainly through the application of spatial analytical methods such as buffer analysis or the construction of Voronoi diagrams; i.e. a spatial partitioning method for identifying the respective territory against a set of origins. For instance, Nakaya [12] constructed a point Voronoi diagram based on the water pump locations as well as a Kernel Density surface to illustrate the concentration of the victim locations around the BSP. Similarly, Koch [3] constructed equidistant buffers around each pump to demonstrate that the BSP was at the centre of where the majority of victims had lived.

While these studies used the Euclidian, straight-line distance in their nearest neighbour analysis, Cliff and Haggett [13] used a network-based Voronoi diagram to identify the area of influence for the BSP by walking distance along the street network. It follows the same principle as Snow’s equidistance line [2] and indeed produced a result that was very similar to what Snow had drawn by hand. Furthermore, Shiode [6] constructed a complete network-based Voronoi diagram using all 13 pumps in the study area, as well as a revised diagram without the Little Marlborough Street pump, which Snow [14] reported as producing unclear water and was underused by the local residents, which ironically promoted the use of water from the BSP, which was actually contaminated.

Most of these studies focus on examining the extent of the BSP’s area of influence and the number of victims accounted for in that area, rather than measuring the mortality rates. This is because information on the number of residents in each house at the time of the outbreak has not been made readily available. Koch and Denike [15] attempted to estimate the mortality rate within each pump’s area of influence by dividing the number of deaths by the number of houses within the respective area, and multiplying each figure by an estimated constant of 10 persons per house. Their study showed that, in the BSP’s area of influence, much higher rates of death per house were observed than that of any other pumps’. However, it was based on the comparison of a single estimated value for each area. In order to examine the mortality rates at a finer scale, we need, ideally, the number of residents for each house in the study area at the time of the outbreak, with which to divide the number of victims in each house. While several studies refer to the scope of using historical census [6], it was never pursued until now. This study attempts to address this gap in the literature by referring to historical records to construct a database of household population in the area as accurately as possible.

The other strand of studies has mainly investigated the point pattern found among the victims’ locations to empirically observe their association with the BSP’s position. For instance, Nakaya [12] applied KDE to produce density surface of the risk of cholera in the study area at the time of the outbreak. Shiode [6] applied a network-based variable clumping method [16] to demarcate the most significant concentration of victim locations and also to explore the distance decay effect on the reduction of the density with the increase in the street network distance from the BSP.

However, these studies did not test for the significance of such clusters, nor did they explore the space-time pattern of the spreading of the disease. One way to detect such clusters and test for their significance is to apply Scan Statistic [17], a cluster detection method commonly used in epidemiology for identifying disease hotspots whilst taking into account the inhomogeneous spatial density. Shiode [8] recently proposed NetScan, a network-based variation of Scan-Statistic type hotspot detection methods, designed for micro-scale analysis of clusters along networks. Furthermore, Shiode and Shiode [18] developed a spatial-temporal network-based hotspot detection method, and Shiode and Shiode [19] extended it as a geo-surveillance system for monitoring emerging hotspots.

As the victim locations are recorded by their street address, use of a network-based cluster detection method, as opposed to the conventional Euclidean distance method, would help improve the understanding of the degree of their concentration. Given the capacity of the network-based methods to improve the accuracy of hotspot detection at the micro-scale level of street address, they would suit the scope of this study well and, while the method proposed by Shiode [8] and Shiode and Shiode [18] use crime data as a test case, their methods are fundamentally applicable to epidemiological studies also. It will be also in keeping with Snow’s original idea of the equidistance line. This study therefore adopts the NetScan to explore how cholera has affected the study area during this period of outbreak at the micro-scale level of street address.

## Methods

In order to derive the estimated mortality rates for each house location and the space-time pattern of disease spreading, a series of historical documents is consulted from which a set of GIS data is prepared. These data are subsequently visualised in map forms, and analysed using KDE as well as NetScan [8]. NetScan is a recently developed method for detecting statistically significant concentrations along street networks to render information for interpreting where and when such events have unfolded. A brief description of KDE and NetScan is provided below.

### Kernel Density Estimation (KDE)

KDE is a density estimation method that uses known recorded values to produce a density surface across the study area. In its simplest form, a circular kernel is formed around each generator point (e.g. locations of victims) with a predefined bandwidth as their radius. The density surface is produced by assigning each location within the study area the sum of these kernels for the respective location. As each dataset and the geography of the study area are unique, the KDE bandwidth is typically decided in an exploratory fashion where KDE is produced using different bandwidth sizes so as to empirically derive a suitable value for that specific context.

### Network-based Scan Statistics (NetScan)

NetScan [8] allows us to identify clusters of events at the micro-scale level of street address. While NetScan is a search-window-type technique similar to the conventional Scan Statistics [17], it uses a sub-network that flexibly adapts to the configuration of the street network, rather than a circular search window, to sweep across the study area for detecting clusters [8]. As it identifies significant concentrations of events with respect to the shortest path distance from the centre of a search window, NetScan is particularly suitable for the purpose of detecting clusters in a small study area where events are confined by the street paths.

The significance of the clusters will be tested against the likelihood ratio; i.e. the calculated likelihood ratio indicates how likely a cluster would have occurred by chance under the given population at risk. Let *N*_*sw*_ be a search window created along the street network. The likelihood ratio for *N*_*sw*_ is:

$$ \begin{array}{c}\hfill \frac{L\left({N}_{sw}\right)}{L_0}={\left\{\frac{n\left({N}_{sw}\right)}{\mu \left({N}_{sw}\right)}\right\}}^{n\left({N}_{sw}\right)}{\left\{\frac{N-n\left({N}_{sw}\right)}{N-\mu \left({N}_{sw}\right)}\right\}}^{N-n\left({N}_{sw}\right)},\ \mathrm{if}\ n\left({N}_{sw}\right)>\mu \left({N}_{sw}\right),\  and\hfill \\ {}\hfill \frac{L\left({N}_{sw}\right)}{L_0}=1,\ \mathrm{otherwise}\hfill \end{array} $$

where *L*(*N*_*sw*_) is the maximum likelihood for *N*_*sw*_, i.e. how likely the observed data are, given the difference in the rate of deaths inside and outside of *N*_*sw*_, *L*_0_ is the likelihood of the observed data under the null model, *N* is the total number of deaths, *n*(*N*_*sw*_) is the number of deaths observed in the search window, and *μ*(*N*_*sw*_) is the population at risk within the search window under the null model. In the form of an equation, the spatial scan statistic *S*_*NT*_ on the network is the maximum likelihood ratio over all possible *N*_*sw*_:

$$ {S}_{NT}={ \max}_{N_{sw\ }}\left\{\frac{L\left({N}_{sw}\right)}{L_0}\right\} $$.

### Data

Despite its popularity, no prior study has investigated the mortality rates or the space-time pattern of the outbreak in Snow’s map. This is mainly because no single dataset was available for the purpose of these analyses.

This study consults a series of historical documents as listed below; they include Snow’s own data and reports [2, 14] as well as historical census, weekly return records on births and deaths, and ordnance survey maps with the aim to create a database of mortality rates and space-time patterns of victim locations. These additional pieces of information obtained were mostly those that had never been processed or added to Snow’s map. These include the number of residents at each house at the time of the outbreak, or the number of residents on each street as well as the number of houses on those streets which, together with the date and place of the cholera victims allowed us to produce an estimated mortality rate for each street or house.

Therefore, the dataset produced in this study not only holds archival value but also possesses significant academic value as it can be utilised for novel research activities in terms of expanding the scope for spatial, temporal and statistical analyses of Snow’s map.

### Historical data

The maps and reports listed below were obtained from the online John Snow Archive & Research Companion [20] and the Wellcome Trust Library. The Weekly Returns of Deaths were retrieved from the National Archives and Her Majesty’s Stationery Office (HMSO), part of the National Archives of the United Kingdom. Most of these documents were scanned images of the pages, and they were digitised by hand to construct the GIS data.

The GIS data are built upon three existing shapefiles that were released online [21]: (1) 578 deaths from cholera recorded in Snow’s map and report, (2) 13 water pump locations, (3) the street network within the parishes of St James, Westminster, and St Anne, Soho.

- **John Snow’s map** [2]
- **Ordnance Survey map of Golden Square and St. Anne’s Place** (City of London, 1875)
- ‘Plan shewing the Ascertained Deaths from Cholera in part of the Parishes of St James and St Anne, Soho during the summer and autumn 1854’ (hereafter called ‘**Frontage map**’), *Appendix to Report of the Committee for Scientific Inquiries in relation to the cholera epidemic of 1854 from the General Board of Health, Medical Council*, pp.96-97
- **John Snow’s report** from 12 December 1854 submitted to Dr. Lankester’s Cholera Inquiry Committee, constituting Part 3 of the *Cholera Inquiry Committees’ Report to the Vestry of St. James, Westminster parish*
- **House-to-House visitation notes** from *Report on the outbreak of cholera in the Sub-Districts of Berwick Street, Golden Square, and St. Anne’s* [22]
- **Weekly Return No.35**, *Births and Deaths in London*, published by the General Register Office for Week ending Saturday, September 2 (HMSO)
- **Weekly Return No.36***, Births and Deaths in London*, published by the General Register Office for Week ending Saturday, September 9 and its **Appendix to the Weekly Return** (HMSO)
- **Weekly Return No.37**, *Births and Deaths in London*, published by the General Register Office for Week ending Saturday, September 16 (HMSO)
- **Tables of House-to-House Visitation** (hereafter called ‘**HHV’**) in the Golden Square Districts, p.323-352 in *Appendix to Report of the Committee for Scientific Inquiries in relation to the cholera epidemic of 1854 from the General Board of Health, Medical Council* (1855)
- **Summary Table of Houses, Population and Mortality, in the Golden Square Districts** from the *Supplement to the Report of the Committee for Scientific Inquiries in Relation to the Cholera-Epidemic of 1854*
- **Cholera Inquiry Committee Table** (hereafter called ‘**CIC**’) in Appendix B of the *Report on the cholera outbreak in the Parish of St. James, Westminster, during the Autumn of 1854 presented to the Vestry by the Cholera Inquiry Committee*, (London: July 1855)

### Preparation of the database

In order to carry out the study, the following datasets were constructed from the historical documents.

### Streets

The Frontage map was used as a base map and was geo-referenced in a shapefile, as it offers a clear picture of the streets, buildings and street address numbers. A total of 132 streets (and three unnamed short alleys) were found within the extent covered by the Frontage map, which were digitised as 327 street segments, as some streets consisted of several segments. Some addresses had their building footprints and the street number on the Frontage map, and these were digitized directly; whereas those missing from the Frontage map were identified by using the locations shown in the Ordnance Survey maps and the number of houses per street listed in the Summary Table. In total, 2,385 addresses were created.

### Population

**Fig. 1:**
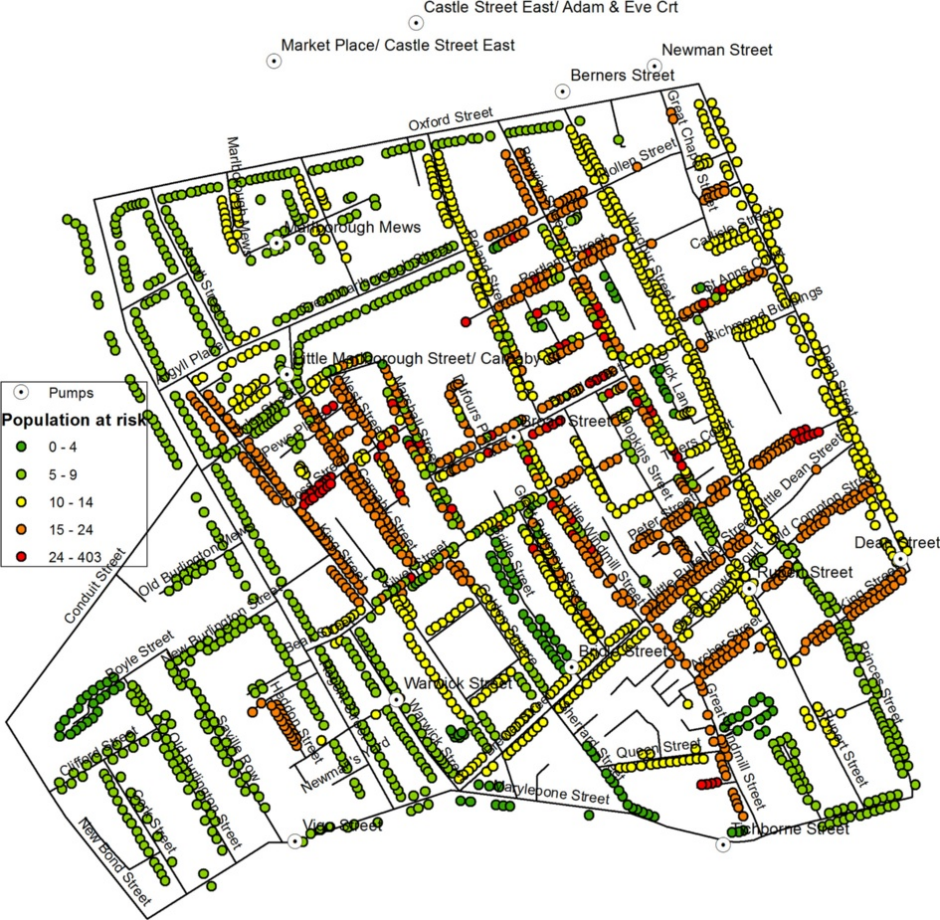
Estimated residential population in each house at the time of the cholera outbreak

The *house-to-house visitations* record showed the number of residents at each street address at the time of the cholera outbreak in August/September 1854. However, they covered only 343 addresses (7 % of the 2,385 addresses), accounting for a population of 4,906. Therefore, the number of residents at the remaining addresses was estimated through a combination of HHV, CIC and Summary Table, which collectively offered (1) the number of houses on each street, and (2) the number of residents on the respective street; thus allowing us to obtain the average number of residents for each street. For instance, in Husband Street, the 120 residents recorded in the CIC were divided between the 10 houses recorded in the Summary Table, assigning an estimated number of 12 residents per house to each address. Figure 1 shows the estimated number of residents at each street address. While this process admittedly introduces some inaccuracy and averages out any variation in the number of residents within each street, it is the best estimate we could make in the absence of the exact figures. Also, non-residential units such as a large factory or a stable were clearly indicated as such, and these were accounted for individually.

### Cholera deaths

**Fig. 2:**
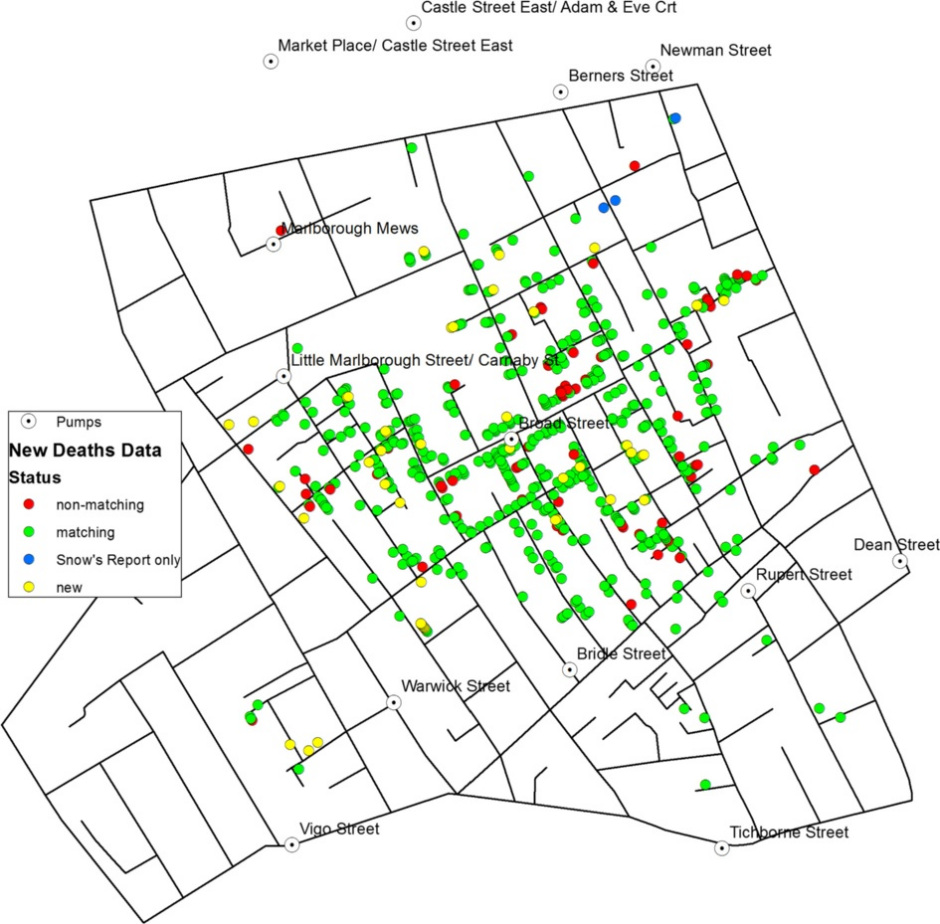
A map of victim locations including additional records

Information on the cholera victims were mainly extracted from the *Weekly Returns* and the *Appendix to the Weekly Return* as well as Snow’s reports [2, 14] which were based on the *Weekly Returns*. Of the 578 records of death on Snow’s map, 509 cases (88 %) were matched to an entry in the *Appendix* or *Weekly Returns*. Among these 509 cases, 409 locations matched perfectly (listed as ‘matching’ in Fig. 2), whereas 100 cases had some discrepancy in their address between Snow’s report and the *Appendix of Weekly Returns* in that they were one or a few street numbers away (listed as ‘non-matching’ in Fig. 2). These were matched to the closest street number on Snow’s map. All but three victims in Snow’s report were found in the *Appendix of Weekly Returns*. In contrast, there were 45 cases that did not show on Snow’s map but were recorded in the *Appendix* and the *Weekly Returns* (listed as ‘new’ in Fig. 2). Between the original records of 578 victims from Snow’s map and the additional 45 cases, a total of 623 deaths are investigated in this study.

### Limitations

It should be noted that, while this dataset was collected and processed to the best of our knowledge, it may be prone to some error, such as misrepresentation of point locations, omission of a point location, or incorrect date entry. Where possible, the data were cross referenced between multiple sources listed herewith, and the margin of error, if it exists, would be not significant and is bound by the level of accuracy of the resources. In terms of their impact on the outcome, KDE would be less susceptible to such error as its bandwidth would carry over an extended area, whereas NetScan could suffer from omission or misrepresentation of point locations, and this would have been reflected in the results obtained below.

Also, the dataset prepared in this study does not account for the difference in the age of victims or that of population at risk, whereas the risk may have in fact varied between different age groups. For instance, the elderly were reported to have less cholera incidence than younger persons, as they lived on upper floors, thus having poor access to the water from the pump, and tended to consume other types of drink such as wine and beer [23].

Another point to note is that not all deaths may have been caused through direct consumption of contaminated water. Some cases may have resulted from a secondary infection; i.e. food or close contact with someone already infected. This second wave would have emerged after an incubation period where the first wave contracting cholera through the BSP subsequently infected others. However, given that the BSP was in operation for some time, these two waves would have overlapped with one another. While the second wave consists of acquaintances of the BSP users, their proximity to the BSP can be only inferred. Some cases from the second wave may be found within the proximity of the BSP but were contracted indirectly, while other cases may be farther away from the BSP location. As there is no record of how the victims contracted the disease, the former category will be interpreted as part of the first wave, while the latter will be detected as anomalies, which may account for a portion of the cases outside the area that the BSP served. In terms of the outcome of the analysis, this may not have a significant impact, but it is important to note the composition of these cases.

Finally, use of the cholera mortality data for measuring etiological parameters needs attention. Given that the etiological link is with the time, place and cases of infection, rather than with the time, place and cases of death, there is a limit to what the mortality data can capture. While it is difficult to estimate the case-fatality among people in London in 1854, figures from the recent cholera experience in Haiti in 2010 suggest that they may have been as low as 5 % [24]. While this inherent limitation applies to all research conducted on Snow’s data, it is worth noting that those who contracted cholera but recovered are not shown on the map.

## Results

### Space-time visualisation

**Fig. 3:**
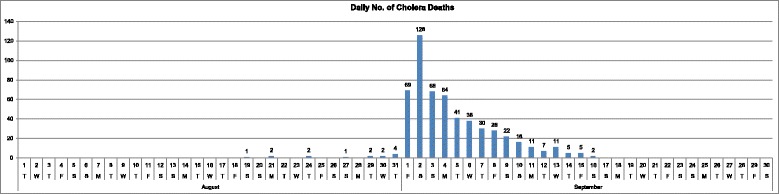
Daily number of cholera deaths (August-September 1854)

Figure 3 shows the daily number of death toll from the cholera outbreak in the study area during August and September 1854. It is known to have started on 28th August 1854 (hereafter called **Day 1**). The initial number of victims was low but increased dramatically in the first week of September. These changes in the number of the daily and weekly death toll can be mapped to see in detail where and when the disease spread.

**Fig. 4:**
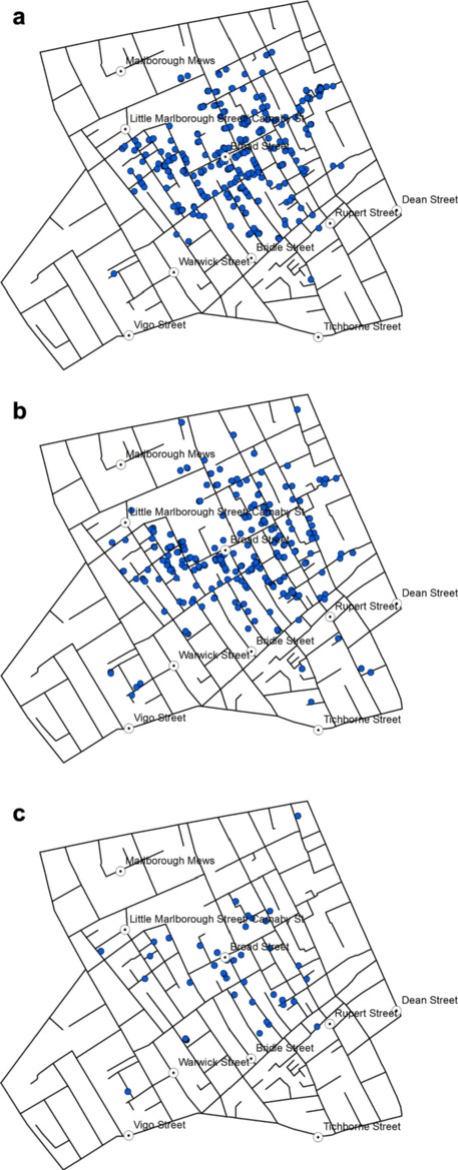
Location of victims reported in **a** Week 3, **b** Week 4, and **c** Week 5

Most of the fourteen cholera deaths recorded in August (of which eight were recorded between 28 August and 1 September) are located on the western part of Soho and three are found farther away in the South. The death toll rises dramatically as we enter the month of September, with 263 deaths having been recorded between 1 and 3 September. Figures 4(a-c) represent the weekly death tolls in Weeks 3 to 5 of the outbreak.

With 271 deaths, Week 3 is the most affected week (Fig. 4a), and the distribution reveals that most of those deaths recorded during that week occurred around Broad Street pump. The death toll continues to remain high during Week 4 (Fig. 4b), with 239 deaths but the distribution is generally consistent with that observed in Week 3. In Week 5, the number falls to 41, but still follow a similar distribution (Fig. 4c). In other words, there is no identifiable space-time pattern that shifts from one area to another over time.

**Fig. 5:**
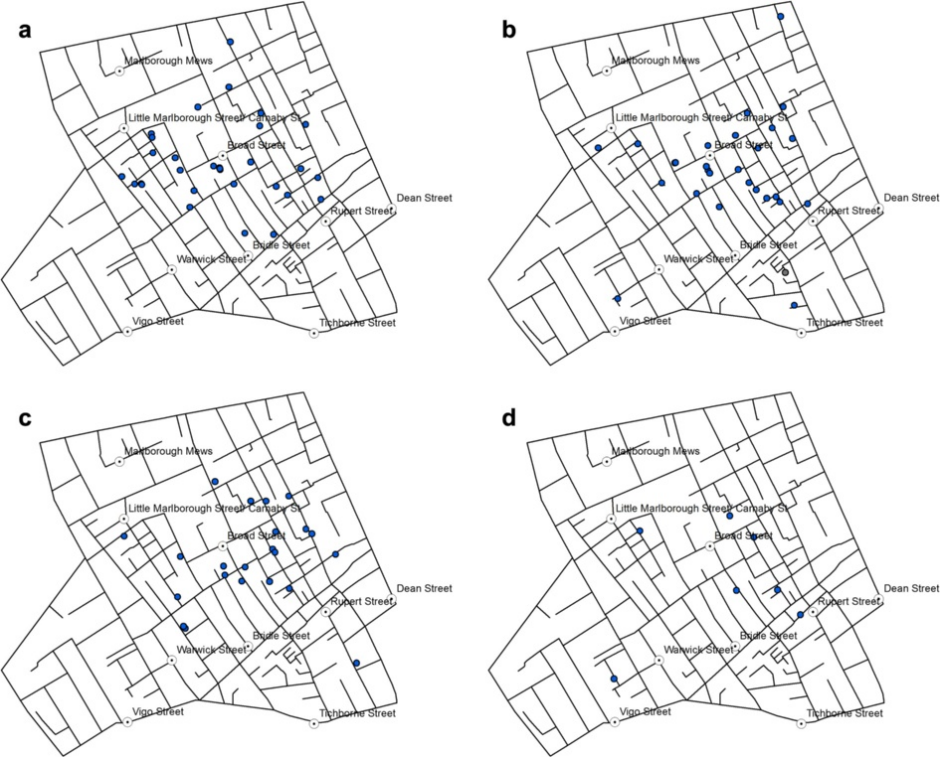
Death from cholera recorded in the study area on **a** 7 September; **b** 8 September, **c** 9 September, and **d** 12 September 1854

Figure 5(a-d) show the location of victims mapped on a daily basis around 7–9 and 12 September. Around this time, a decision was made to remove the Pump handle so as to prevent any further spreading of cholera. The toll falls from 28 deaths on 8th September to 7 deaths on 12th. Without mapping every single day between 5th and 16th September we can compare the distribution before and after the handle was removed to see if some patterns can be detected.

Initial reaction to the removal of the pump handle is less obvious and there is no significant pattern. However, the three deaths recorded three days later on 12th September (Fig. 5d) clearly shows that the victim locations are becoming sparse and spread across the study area, i.e. away from the Broad Street pump.

### Density estimation of the cholera deaths

**Fig. 6:**
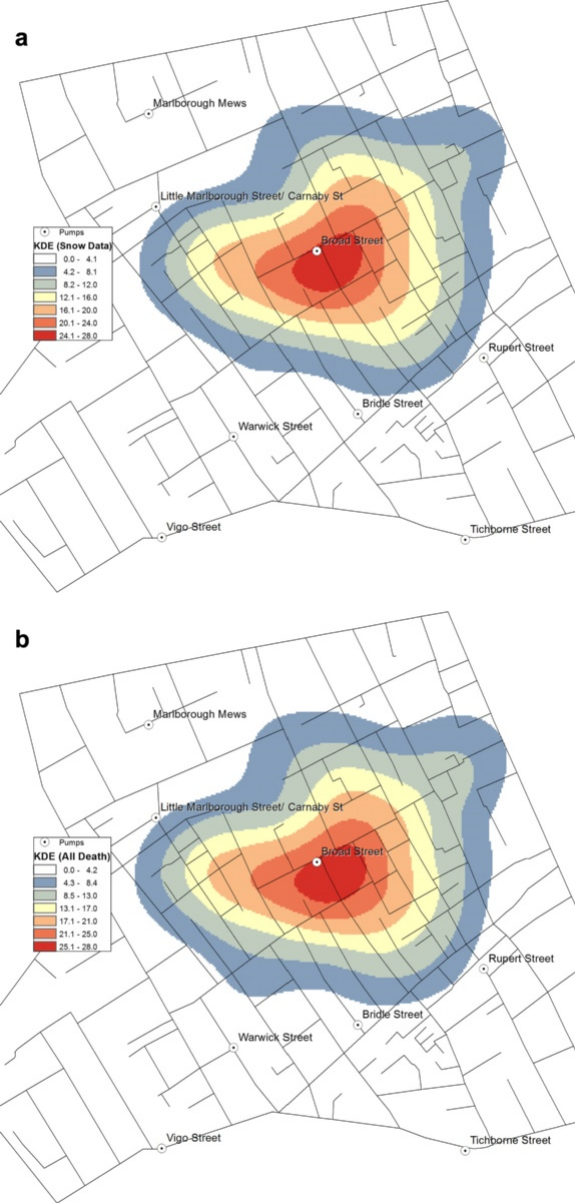
KDE of the cholera death: **a** KDE using Snow’s original data (578 cases); **b** KDE using the new data (623 cases)

Using the space-time data, KDE was also produced for different periods, starting with the entire record consisting of 623 points in total (Fig. 6b). KDE was also created with the 578 cases recorded by Snow in his own map (Fig. 6a).

Comparing Fig. 6(a and b) suggests that the two density estimations are similar and the zone with the highest density of cholera is almost identical. The high density is more prominent on the west side of the area; however, the BSP remains at the epicentre, even with the addition of further 45 deaths.

### Space-time change in kernel density estimation

Using the temporal information of the victim locations, changes in the KDE was explored so as to observe the weekly progression of the clusters of victims. Since Weeks 1 and 2 only witnessed one death and five deaths, respectively, the KDEs were produced for Weeks 3 ~ 5 only.

**Fig. 7:**
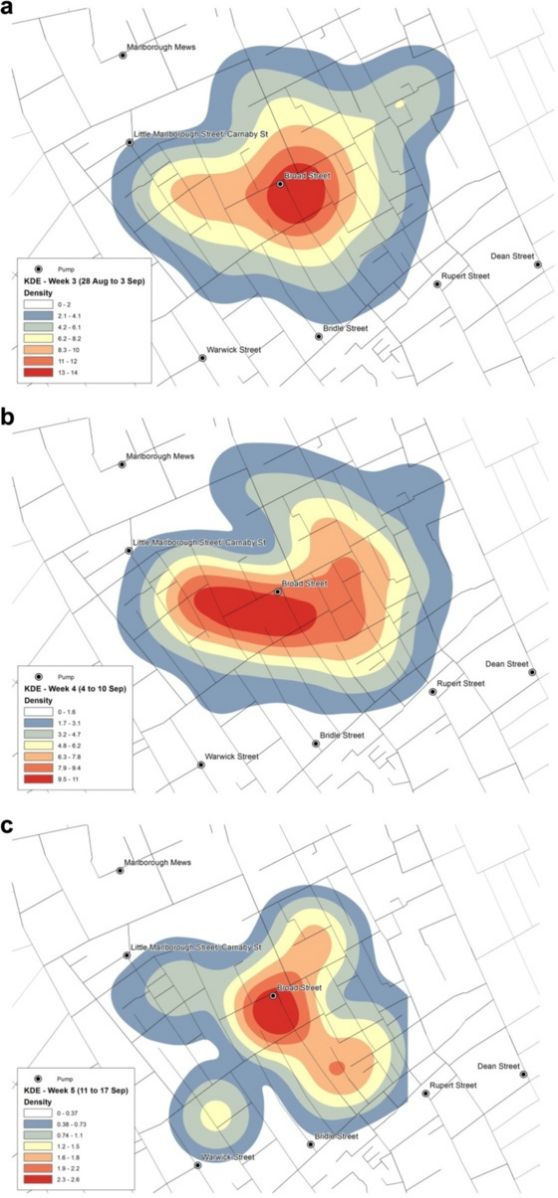
Spatial-temporal KDE: **a** Week 3, **b** Week 4, and **c** Week 5

While Weeks 3 and 5 (Fig. 7(a and c)) share a similar pattern with a strong concentration around BSP, Week 4 sees a departure from this pattern (Fig. 7b). The highest density of deaths starts in Week 3 (Fig. 7a) around the BSP location and towards the east of BSP, whereas in Week 4 (Fig. 7b), the cluster spreads out towards the southerly and south-westerly direction which, in Week 5, is yet again gone and the space-time pattern returns to the state of Week 3. By this time, the estimated overall density estimation is smaller, since there are fewer deaths, and the highest density is found mainly towards south of the BSP location.

### NetScan and network clusters

**Fig. 8:**
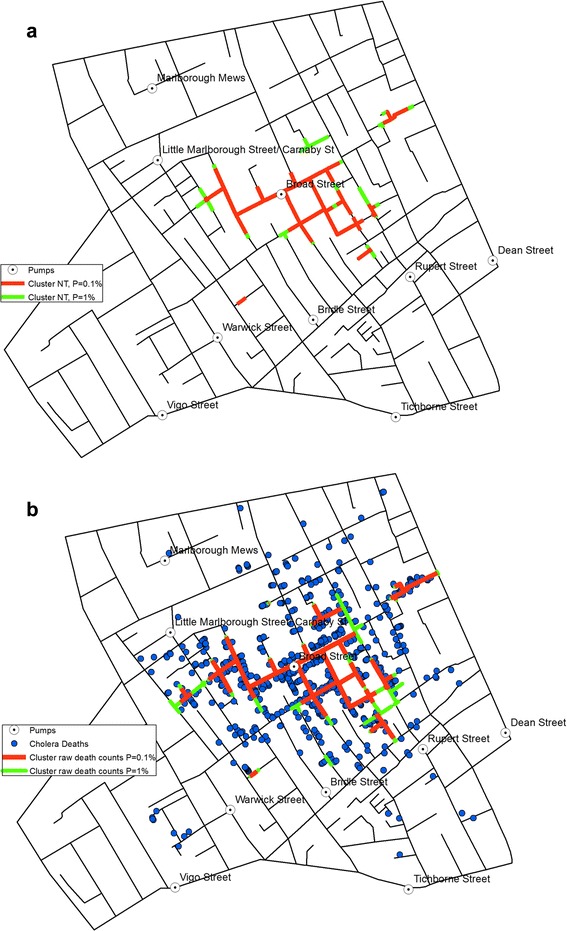
**a** Network clusters based on population at risk, and **b** Network clusters based on death counts

While KDE offers a smooth density surface, it does not provide a detailed account of where the clusters of victim locations are. These clusters can be detected with the application of NetScan instead. Fig. 8a shows such clusters identified through simulations of 999 runs. The red and green lines with high death incidence are derived from the death counts in proportion to the population at risk, and the *p*-value indicates how unlikely it would be for this outcome to happen randomly. The streets in red are the lines detected at 0.1 % significance level, i.e. the most likely clusters. The green lines represent the streets detected with 1 % significance level, the second most likely clusters. NetScan detected strong clusters on streets in the central area as well as on some small isolated street segments in the south, southeast and east.

Figure 8b shows the clusters detected through the measurement of the raw death counts only. Compared with those detected with respect to the population at risk (Fig. 8a), the clusters in Fig. 8b cover a larger area, thus demonstrating that relying on raw counts alone could lead to inaccurate representation of the mortality rates.

The small red segment detected at the workhouse location in Fig. 8b makes a perfect example of how the population at risk affects the outcome. Of the 403 people recorded in the shapefile to be resident at the workhouse, only seven deaths were reported, thus resulting in a cluster of high death counts (Fig. 8b) but low mortality rate that remains undetected (Fig. 8a).

## Discussion

Results of the analyses largely support Snow’s observation on the BSP being ‘in the centre of the area in which the mortality from Cholera occurred’ [2], and this is despite that the data used include additional 45 cases besides the 578 cases reported by Snow.

### Concentration of victims represented through the mortality rates

Indeed, the mortality rates were found to be higher around the BSP. Application of KDE returned density surfaces with their peak formed around the BSP location, which showed a clear tendency of distance-decay in the mortality rate as the distance from the BSP increased. While their patterns were nuanced and generalised, they confirmed that the closer the house stood to the BSP, the greater was the risk of cholera death.

NetScan provided a more precise presentation of the cluster of locations with high mortality rates at the micro-scale level of street address, which again was found mainly around the BSP location (Fig. 8(a and b)). The fact that the outcomes were very similar and consistent between those derived with significance levels set at *p* = 0.001 and *p* = 0.01, respectively, demonstrates the robustness of the cluster formation in the vicinity of the BSP. It is not surprising to see that the clusters derived from the raw counts mainly spread widely across the area originally demarcated by the equidistance line (Fig. 8b), as this was what Snow had originally observed as the area with high concentration of victims. In contrast, the clusters detected through the examination of the mortality rates formed a clearer and more compact concentration in the vicinity of the BSP, thus presenting even stronger evidence that the water from the BSP was to blame for the disease outbreak. It implies that, within the area identified by Snow’s equidistance line (i.e. the area for which the BSP is considered to be the closest water pump than any other pumps are), there was a difference in the amount of consumption of the contaminated water from the BSP; i.e. the contamination and the higher risk were more prominent in the immediate vicinity of the BSP, whereas areas in the periphery of the equidistance line showed a somewhat lower mortality rate (Fig. 8a), thus implying that some of the households in those areas may have been accessing other water pumps, which provided uncontaminated water.

Some small isolated street segments in the south, southeast and east were also found to have clusters of high victim counts and this was also reflected in the detection of higher mortality rates in those streets. These can be mainly attributed to the presence of a small number of households suffering from a large number of victims which pushed up the overall count of victims in a small segment of the respective street.

### Space-time pattern of the disease spreading

The space-time visualisation (Figs. 3, 4 and 5) also demonstrated the centrality of the BSP, and this was particularly visible in the weekly distribution during the two weeks from 28th August to 10th September (Weeks 3 and 4 shown in Fig. 4(a, b)). While they did not reveal any clear sign of the disease spreading in a specific direction over time, the variation in the centre of the KDE observed between Weeks 3 to 5 (Fig. 7a-c) gave a more dynamic account of the outbreak in that the centre of the high risk locations shifted around BSP over the three-week period.

The reduction in the number of victims in Week 5 is also consistent with the timing of the disruption of water supply from the BSP. Following Snow’s meeting with the Board of Guardians of St. James’s parish on 7 September where he presented the result of his investigations, the pump handle for the BSP was removed on 8 September to prevent further consumption of the contaminated water by the local residents. While the removal of the pump handle did not immediately stop the outbreak, the peak of the epidemic had subsided by then. Snow also noted ‘the daily number of fatal attacks was already much diminished by 8 September [2]. However, distribution of victims in Week 5 starting on 12 September (Fig. 5d) suggests that they were no longer centred on the BSP.

It should be noted, however, that given the incubation period of a few hours to 6 days, along with a time-to-death period that ranges from 2 h to 8 days, it is possible that some cases attributed to the BSP may have been still present up to two weeks after the removal of the pump.

### Comparing results of KDE and NetScan analysis

**Fig. 9:**
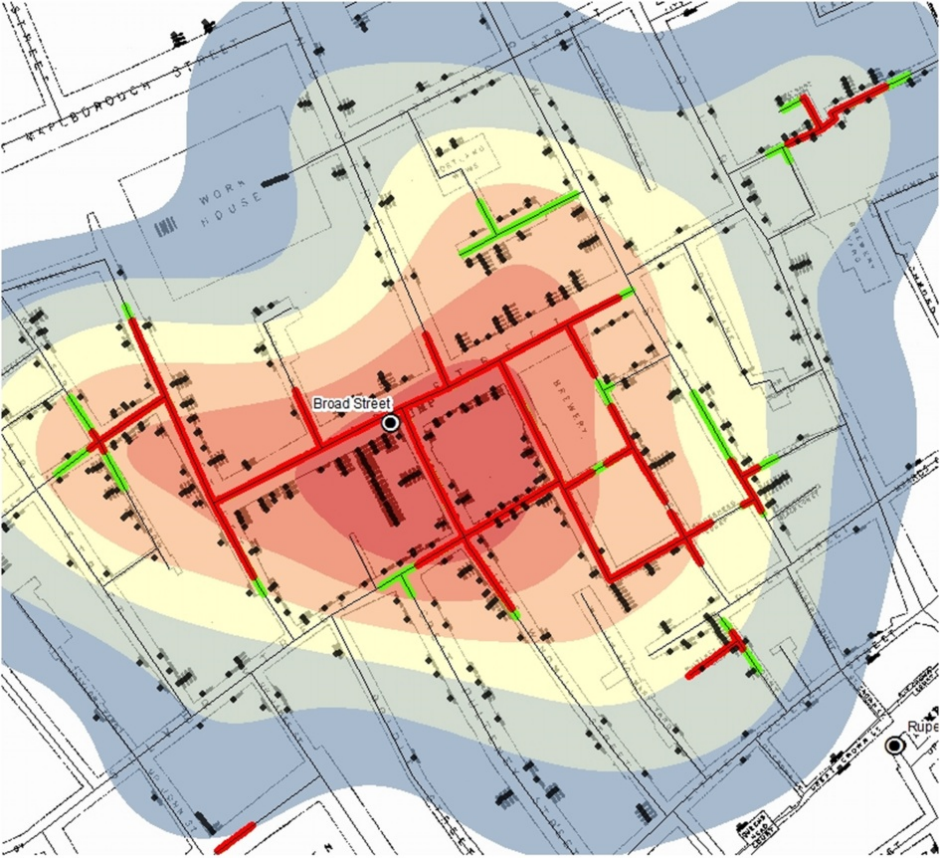
An overlay of KDE and the clusters detected through NetScan

The results produced through KDE and NetScan exhibit different patterns (Fig. 9). The patterns of concentrations detected through the two methods are different in that KDE produces the kernels at each death location, whereas the NT search window moves along the street network. However, Fig. 9 also shows that both methods detect high concentration of victims around the BSP.

Figure 9 also confirms the high concentration of victims around the BSP described in Snow’s Report: ‘It may be noticed the deaths are most numerous near to the pump in Broad Street, where the water could be more readily obtained … The wide open street in which the pump is situated suffered most, and next the streets branching from it, especially those parts of them which are nearest to Broad Street’ [2].

Snow adds: ‘If there have been fewer deaths in the south half of Poland Street than in some other streets leading from Broad Street, it is no doubt because this street is less densely inhabited’. Figure 1 confirms that the population was indeed estimated to have been low in the southern section of Poland Street. This is also reflected in Figs. 8a and 9 where NetScan shows no significant clusters in Poland Street, especially around the workhouse location, whereas the clusters detected from the raw counts (Fig. 8b) draws a cluster in that area without accounting for the lack of population at risk.

Both methods used in this study provide effective means of visualisation for examining the source and spread of cholera. They respectively transform the object of investigation, deaths, into different patterns in that KDE (Fig. 6(a and b)) provides a lens to observe the association between location of deaths due to cholera in relation to that of the BSP; whereas NetScan provides a lens to examine clusters of deaths (Fig. 8(a and b)). Together, they support a source of contamination of water at the BSP, especially as NetScan shows several clusters of deaths there is not likely a local airborne source (Fig. 9). In this sense, these methods are particularly useful in exploring a single source outbreak in a local situation with the dataset being used as a bench for modeling.

## Conclusions

This study re-examined Snow’s map by investigating the mortality rates and the space-time pattern of the spreading of the epidemic. They were made possible by constructing datasets that incorporate victim locations from Snow’s and other reports, the number of household residents, and the date of death for each victim. It showed that houses around the BSP’s location suffered from high mortality rates, thus confirming the water from the BSP to be the source of contamination. The study also carried out spatial-temporal analysis of the victim distribution to examine how the epidemic has spread across the area over time. In general, there was no discernible pattern to indicate the spreading of the disease in a specific direction.

Despite the numerous existing studies on Snow’s work, no previous effort was found in understanding the mortality rate or the space-time pattern of the outbreak at the disaggregate, micro-scale and, therefore, the new digitised information on individuals, addresses and population can be considered as an original contribution in its own right. While the exact number of household population was available for only a subset of the entire study area and the rest were estimated from the total number of residents recorded for each street in the immediate previous census taken in 1851, this additional data of residents and the estimated mortality rates open an entirely new facet for statistical analysis, as they offer better grasp of the actual risk for each household location.

The other dataset constructed in this study was the list of dates of deaths and the address locations of the victims’, which also covered a greater number of victims than was recorded in Snow’s map. While they did not reveal any new patterns of interest, application of KDE to the new dataset revealed a hotspot similar to that derived from the original data. Also, the weekly and the daily KDEs were effective in showing the displacement of highest density of death counts.

Using the known and the estimated numbers of residents, the clusters detected along the street network were confirmed in the BSP’s area of influence, but gave a more robust statistical outcome than was previously studied. The results obtained were largely consistent with Snow’s findings in that the main cluster was found within the extent of Snow’s equidistant line, and only few outside clusters were newly identified, which are primarily attributed to the additional historical reports used in constructing the new dataset.

Adding more detailed information did not fundamentally change the outcome of Snow’s original work, but it allowed us to appreciate the considerably high mortality rates observed in the vicinity of BSP’s, and the pattern of evolution of the disease.

The study did not conclusively eliminate competing theories of the day (e.g. a multifactorial explanation) to confirm Snow’s view of cholera as being solely waterborne. However, the outcome of NetScan analysis provides sufficient evidence, at a micro-scale of the study area, to assume the presence of a local source, rather than cholera being airborne. In order to confirm the sole responsibility of a single source, a study covering a larger urban setting of London in 1854 would be desirable, which would further compound the interpretation of Snow’s theory in terms of the single source and local venue of the outbreak as well as plausible alternative sources outside the study area.

## Abbreviations

BSP: Broad Street pump
KDE: Kernel Density Estimation
NetScan: Network-based Scan Statistic
