# Erratum to: The mortality rates and the space–time patterns of John Snow’s cholera epidemic map

**DOI:** 10.1186/s12942-015-0016-6

**Published:** 2015-11-16

**Authors:** Narushige Shiode, Shino Shiode, Elodie Rod-Thatcher, Sanjay Rana, Peter Vinten-Johansen

**Affiliations:** Centre for Interdisciplinary Methodologies, University of Warwick, Coventry, CV4 7AL UK; Department of Geography, Environment and Development Studies, Birkbeck College, University of London, Malet Street, London, WC1E 7HX UK; Department of History, Michigan State University, East Lansing, MI 48824-1036 USA

## Erratum to: International Journal of Health Geographics (2015) 14:21 DOI 10.1186/s12942-015-0011-y

The original version of this article unfortunately contained mistakes. Figures 1, 2, 3, 4, 5 and 8 should have been revised figure files provided by the author. The figures were not updated with the revised image files. The original article was corrected.

The correct versions of Figs. 1, 2, 3, 4, 5 and 8 are below:

**Fig. 1:**
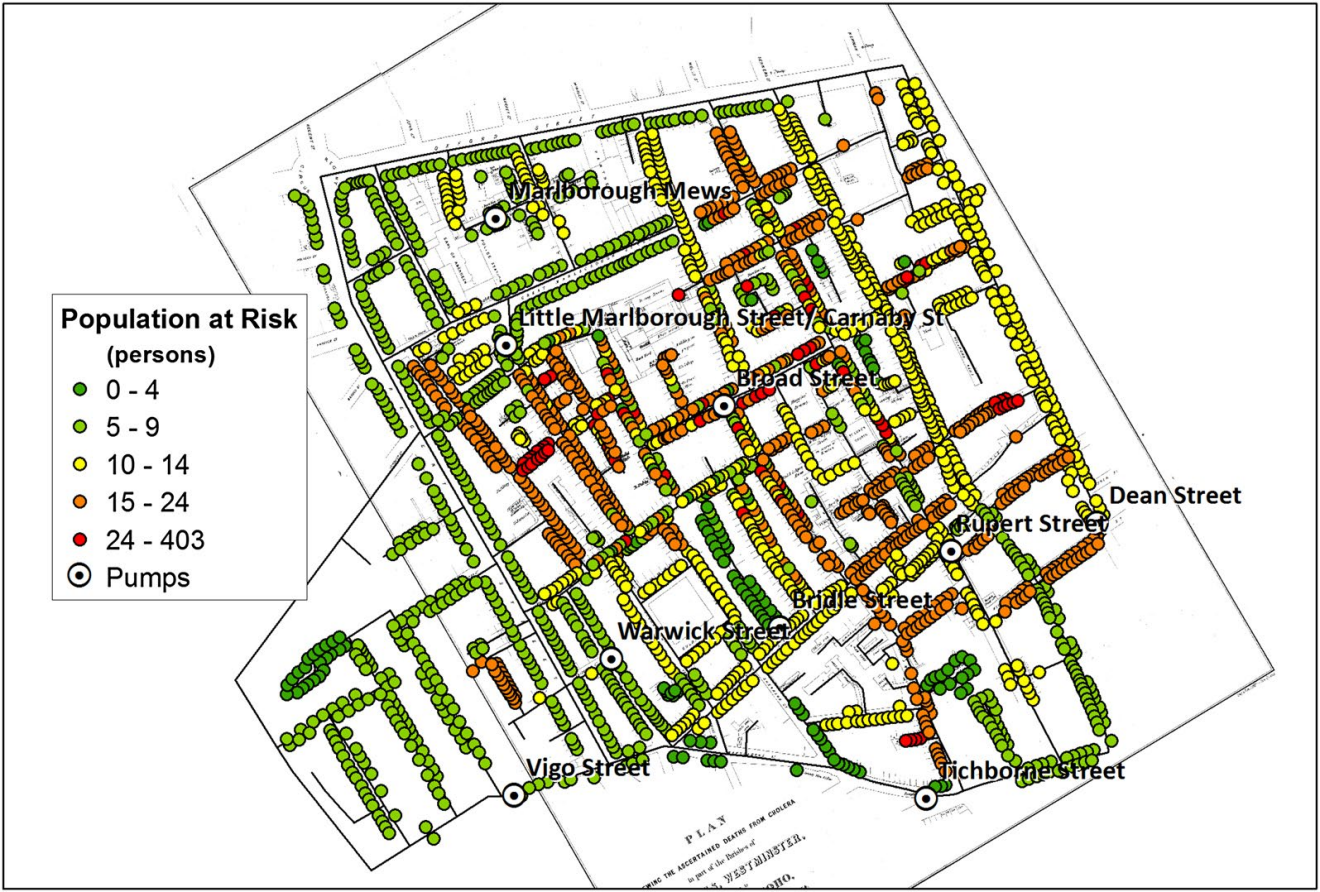
Estimated residential population in each house at the time of the cholera outbreak

**Fig. 2:**
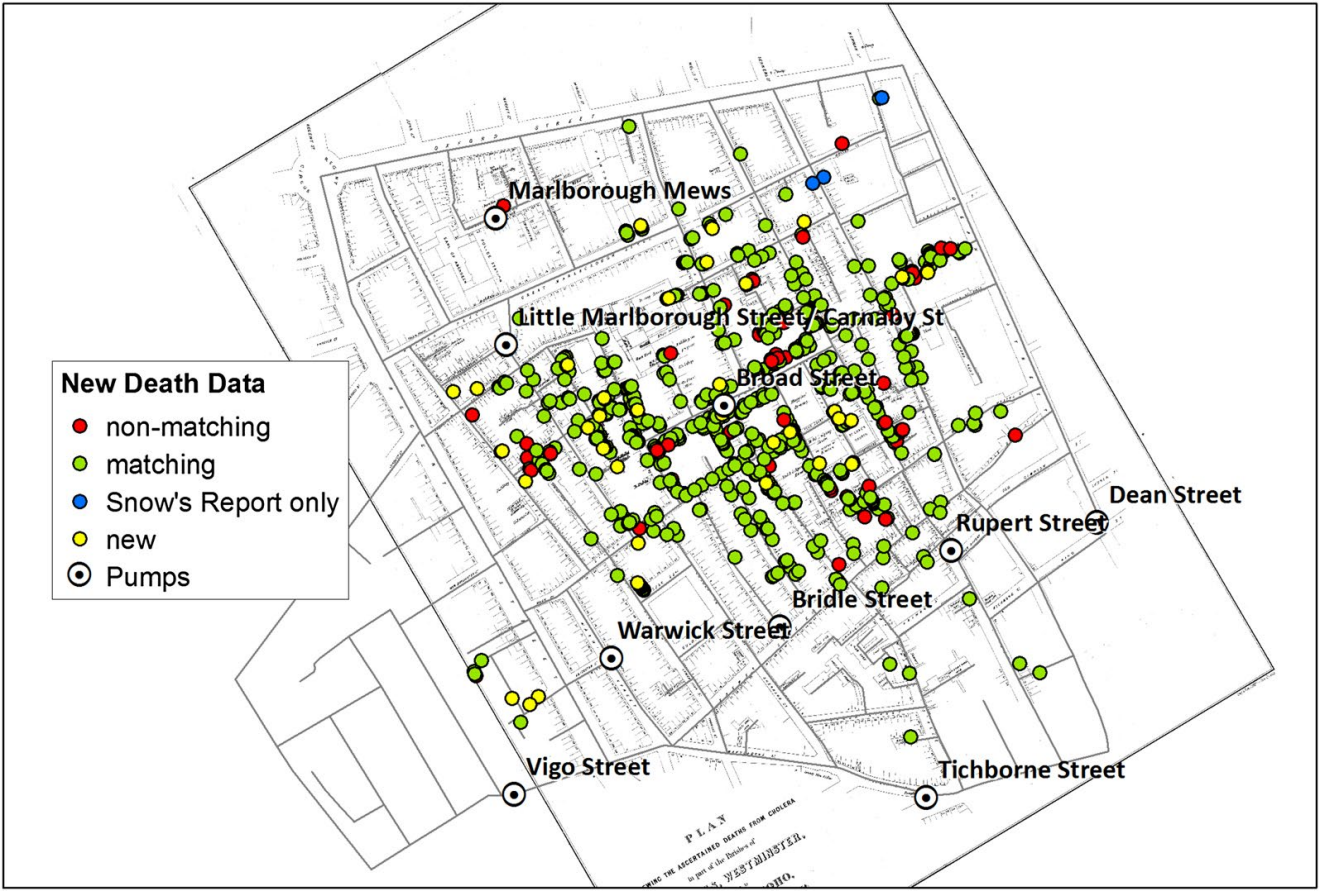
A map of victim locations including additional records

**Fig. 3:**
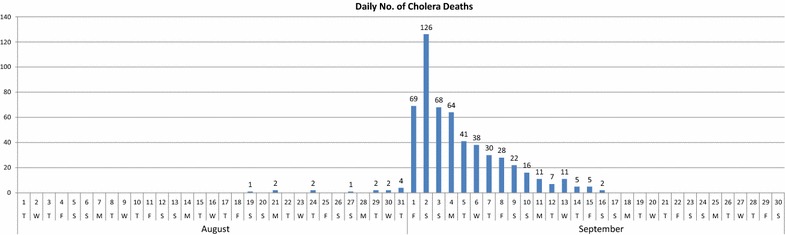
Daily number of cholera deaths (August–September 1854)

**Fig. 4:**
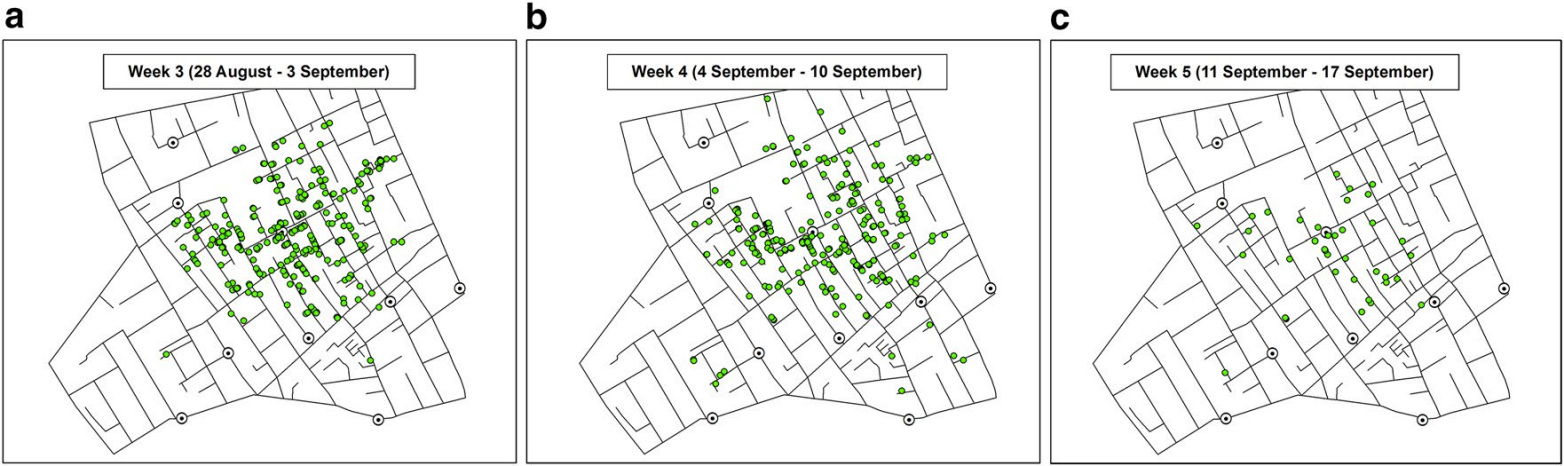
Location of victims reported in **a** Week 3, **b** Week 4, and **c** Week 5

**Fig. 5:**
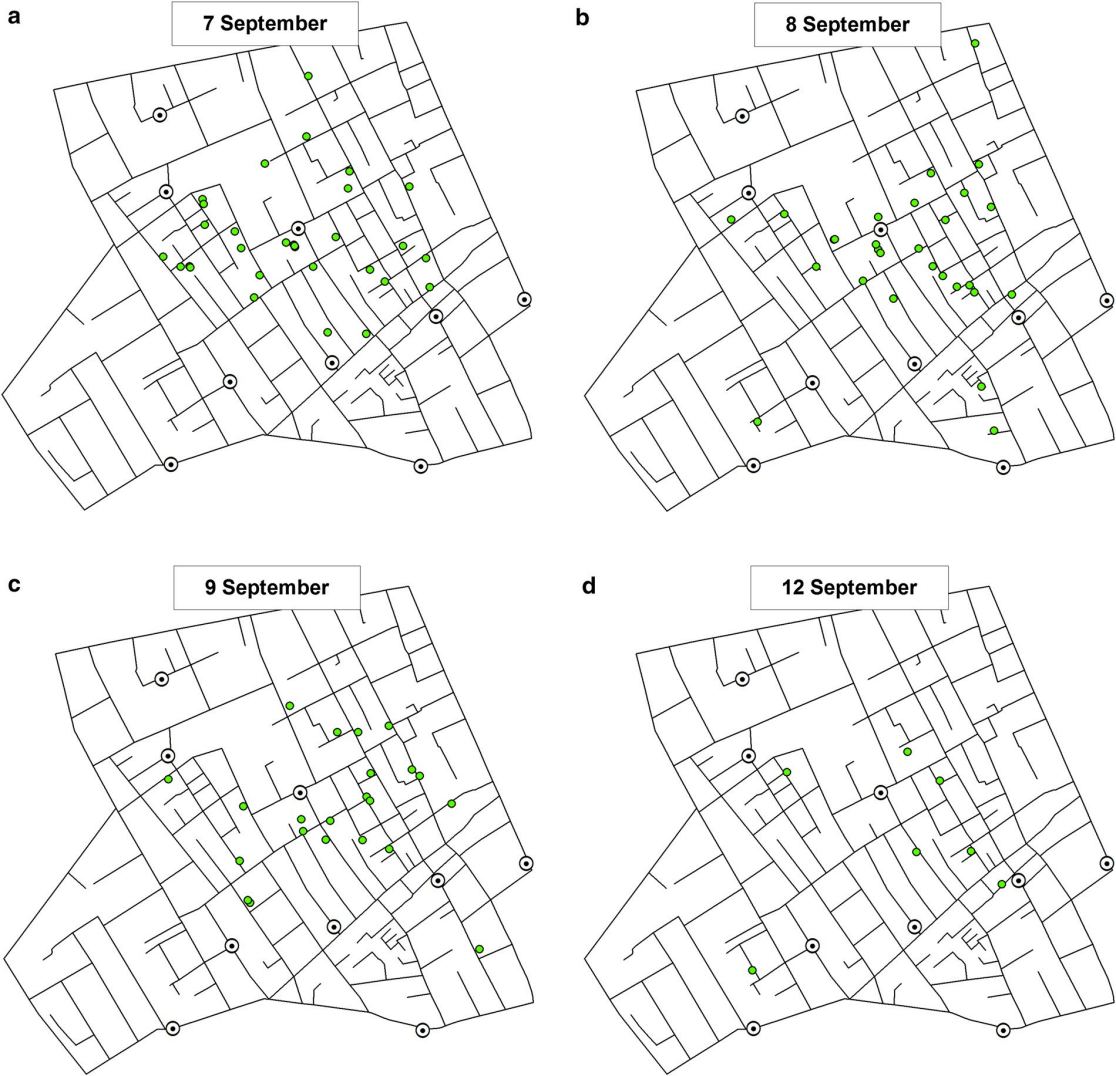
Death from cholera recorded in the study area on **a** 7 September; **b** 8 September, **c** 9 September, and **d** 12 September 1854

**Fig. 8:**
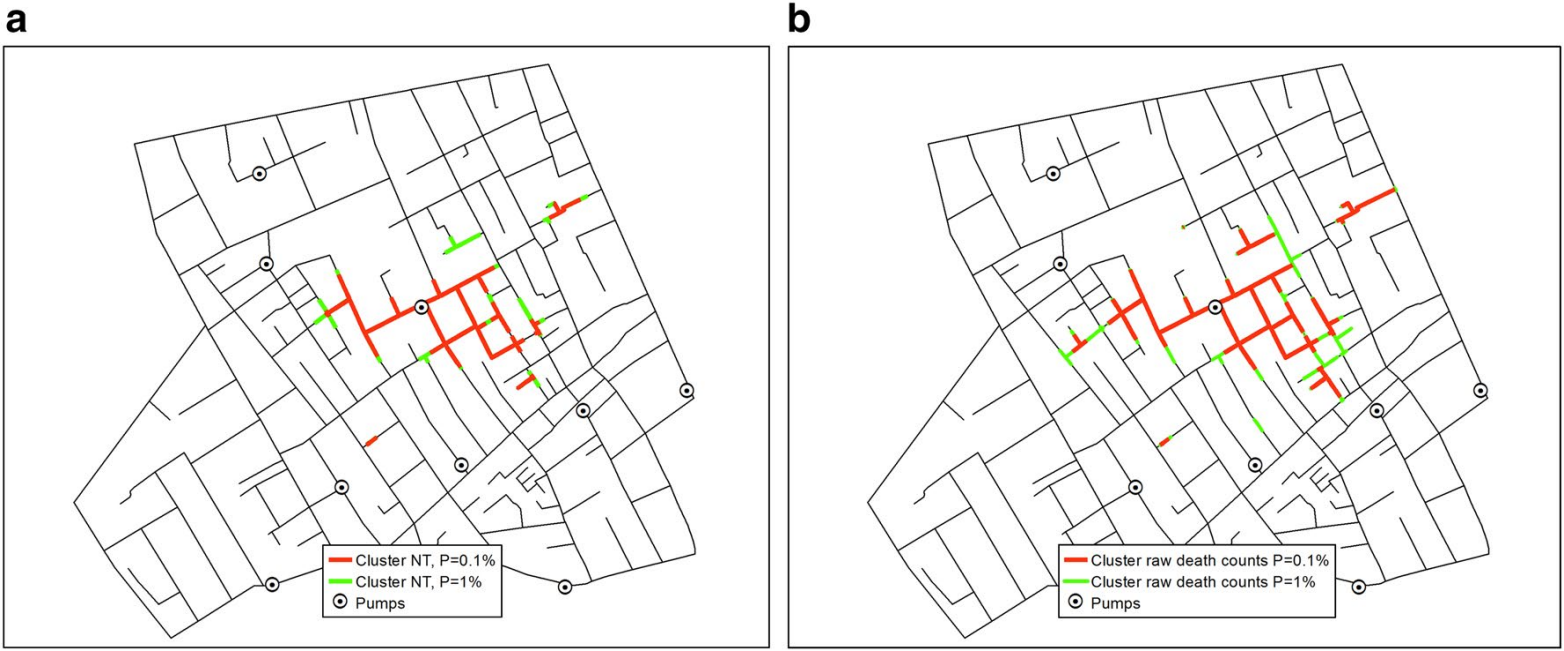
**a** Network clusters based on population at risk, and **b** network clusters based on death counts

